# Unsuspected pyocyanin effect in yeast under anaerobiosis

**DOI:** 10.1002/mbo3.142

**Published:** 2013-12-05

**Authors:** Rana Barakat, Isabelle Goubet, Stephen Manon, Thierry Berges, Eric Rosenfeld

**Affiliations:** 1 Université de La Rochelle-CNRS-UMR 7266-LIENSs – LIttoral ENvironnement et Sociétés-Team: Approches Moléculaires: Environnement, Santé – Microbial Physiology GroupAvenue Michel Crépeau, 17042, La Rochelle Cedex 1, France; 2 Institut de Biochimie et Génétique Cellulaires-1rue Camille Saint Saëns, 33077, Bordeaux Cedex, France; 3 Université de Poitiers-Institut de Physiologie et Biologie Cellulaires (IPBC) – CNRS FRE 3511 – Pôle Biologie Santé-1Rue Georges Bonnet, 86022, Poitiers, France

**Keywords:** Aerobiosis, anaerobiosis, oxidative stress, phenazine, pyocyanin, *Saccharomyces cerevisiae*, yeast mutants.

## Abstract

The blue–green phenazine, Pyocyanin (PYO), is a well-known virulence factor produced by *Pseudomonas aeruginosa*, notably during cystic fibrosis lung infections. It is toxic to both eukaryotic and bacterial cells and several mechanisms, including the induction of oxidative stress, have been postulated. However, the mechanism of PYO toxicity under the physiological conditions of oxygen limitation that are encountered by *P. aeruginosa* and by target organisms in vivo remains unclear. In this study, wild-type and mutant strains of the yeast *Saccharomyces cerevisiae* were used as an effective eukaryotic model to determine the toxicity of PYO (100–500 *μ*mol/L) under key growth conditions. Under respiro-fermentative conditions (with glucose as substrate), WT strains and certain H_2_O_2_-hypersensitive strains showed a low-toxic response to PYO. Under respiratory conditions (with glycerol as substrate) all the strains tested were significantly more sensitive to PYO. Four antioxidants were tested but only N-acetylcysteine was capable of partially counteracting PYO toxicity. PYO did not appear to affect short-term respiratory O_2_ uptake, but it did seem to interfere with cyanide-poisoned mitochondria through a complex III-dependent mechanism. Therefore, a combination of oxidative stress and respiration disturbance could partly explain aerobic PYO toxicity. Surprisingly, the toxic effects of PYO were more significant under anaerobic conditions. More pronounced effects were observed in several strains including a ‘petite’ strain lacking mitochondrial DNA, strains with increased or decreased levels of ABC transporters, and strains deficient in DNA damage repair. Therefore, even though PYO is toxic for actively respiring cells, O_2_ may indirectly protect the cells from the higher anaerobic-linked toxicity of PYO. The increased sensitivity to PYO under anaerobic conditions is not unique to *S. cerevisiae* and was also observed in another yeast, *Candida albicans*.

## Introduction

Pyocyanin (PYO, 1-hydroxy-N-methylphenazine) is a blue–green redox-active secondary metabolite produced by the common opportunistic pathogen *Pseudomonas aeruginosa*. This pigment is an important virulence factor (Lau et al. 2004a,2004b; Caldwell et al. 2009; Hunter et al. 2012). It is toxic to a broad range of target organisms including bacteria, yeast, and mammalian cells (Hassan and Fridovitch 1980; Baron and Rowe 1981; Kerr et al. 1999; Muller 2006).

**Figure 1:**
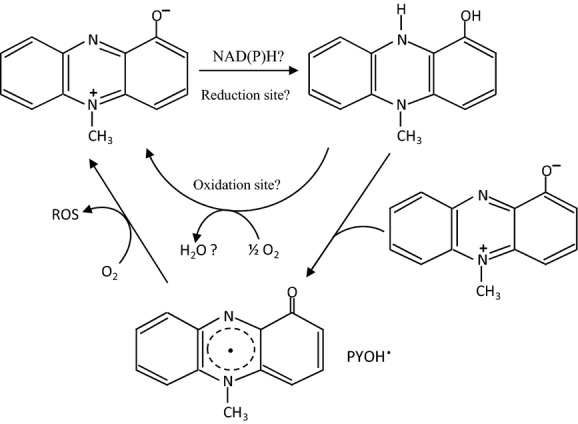
Hypothetical pyocyanin redox cycling (adapted from Jacob et al. 2011). Oxidized pyocyanin (blue) can be reduced nonenzymatically by NAD(P)H. The reduced pyocyanin (colorless) reacts with the oxidized form to generate the highly reactive pyocyanin radical (PYOH^·^). In the presence of O_2_, the auto-oxidation of pyocyanin leads to reactive oxygen species (ROS) production (O_2_, H_2_O_2_). Both oxidized and reduced pyocyanin may interfere with the respiratory chain.

Many of the cytotoxic effects of PYO result from its ability to undergo a redox cycle (Gloyne et al. 2011; Fig. 1). PYO can be reduced nonenzymatically by NAD(P)H and this reduced form can react with O_2_ to produce superoxide (O_2_^·−^), and by dismutation, hydrogen peroxide (Hassan and Fridovitch 1980; David and Thornalley 1983; Müller et al. 1989; Britigan et al. 1992; Muller 2011). Moreover, it can affect host antioxidant mechanisms by inactivating catalase (CAT) and depleting glutathione levels (Muller 2002; O'Malley et al. 2003a). It is noteworthy that PYO directly reacts with several fluorescent probes (e.g., 2′,7′-dichlorodihydrofluorescein, dihydrorhodamine), which are commonly used to detect reactive oxygen species (ROS) (O'Malley et al. 2004b). Data correlating oxidative stress with PYO toxicity should therefore be taken with caution. It has also been suggested that PYO interferes with the respiratory chain and diverts electrons from it, potentially lowering cellular ATP content (Friedheim 1934; Armstrong and Stewart-Tull 1971; Baron et al. 1989). O'Malley et al. (2003b) showed that the addition of PYO has a dramatic impact on the ultrastructure of mitochondria from epithelial lung cells and these authors suggested that the redox cycling of PYO takes place in or near mitochondria. However, despite numerous studies, the subcellular site(s) of PYO redox cycling and the mechanisms underlying PYO toxicity remain unclear. Data published on the toxic effects of PYO in eukaryotic cells were performed under aerobic conditions. The few studies conducted under hypoxia or anaerobiosis were on bacteria (Hassan and Fridovitch 1980; Baron and Rowe 1981; Baron et al. 1989; Yoon et al. 2006; Price-Whelan et al. 2007; Wang et al. 2010). Yet, in vivo, both the producer of PYO (*P. aeruginosa)* and the eukaryotic target cells are subject to oxygen limitation, at least at certain periods (Hassett et al. 2002; Worlitzsch et al. 2002; Yoon et al. 2006).

*Saccharomyces cerevisiae* is an eminently suitable model system to study PYO–eukaryote interactions because of its metabolic plasticity and the wide availability of mutants. Ran et al. (2003) used *S. cerevisiae* as a surrogate host to screen and identify mammalian ortholog genes conferring sensitivity to PYO. These authors showed that PYO targets, identified using yeast mutants, in particular, mutations in the vacuolar H^+^-ATPase gene, were also inhibited in human lung epithelial cells. In 2006, Angell et al. assessed the transcriptional effects of PYO in *S. cerevisiae*. However, in both studies, only respiro-fermentative conditions (in YPD medium) with the WT-BY4741 strain (*MAT*a) and derived mutants were used. It should be noted that the WT-BY4741 strain carries a mutation at the *HAP1 locus*, which encodes a major heme-responsive transcription factor that regulates gene expression in response to O_2_ (Gaisne et al. 1999). This strain is impaired in its respiratory capacity and phenomena related to respiration could therefore have been underestimated.

In this study, we examined PYO toxicity in *S. cerevisiae* cells and mitochondria. Different aerobic and anaerobic physiological conditions were used. The effect of PYO was quantified in several strains (including mutants impaired in respiration and/or oxidative stress resistance) under metabolic conditions of oxidation, respiro-fermentation, or pure fermentation.

## Materials and Methods

### Strains

The *S. cerevisiae* strains used in this study were WT-W303 (*MATα*), WT-BY4742 (*MATα*), mutants and constructions derived from WT-BY4742, and others strains differing in pleiotropic drug resistance or affected in DNA damage repair. The RWT-BY4742 strain restored for Hap1p (Gaisne et al. 1999) was also used as WT control. The main characteristics of the strains are summarized in Table S1. Many strains were purchased from EUROSCARF collection. The *erg1Δ*, *hem1Δ*, and *rho*^*0*^ mutants were constructed in the present study. The *erg1Δ* (*TBY24805*) haploid strain was obtained from Y24805 (EUROSCARF) by random spore analysis (Dawes and Hardie 1974) and anaerobic selection on the YPD medium supplemented with ergosterol (80 *μ*g/mL), oleate (1% v/v Tween 80), and geneticin (G418 sulfate) 200 mg L^−1^. This mutant did not grow aerobically and microaerobically on this medium. The *hem1Δ* (*TBY23591*) haploid strain was obtained from Y23591 (EUROSCARF) by random spore analysis and selection on YPD supplemented with *δ*-aminolevulinate 80 mg/L. The *rho*^*0*^ respiratory-deficient mutant was obtained by ethidium bromide induction (Slonimski et al. 1968). We ensured that the strain *rho*^*0*^ was completely mDNA-free by staining the cells with the fluorescent dye, DAPI (4′-6-diamidine-2-phenylindole). For stock culture maintenance and storage, the RWT-BY47442 was grown on yeast nitrogen base (Sigma-Aldrich, Lyon, France) glucose 2% (w/v) devoid of uracil, and the *hem1Δ* mutant was grown in the presence of *δ*-aminolevulinate 80 mg L^−1^ to avoid the enrichment in spontaneous *rho*^*−/0*^ cells.

One strain of *Candida albicans* was used in this study, namely *C. albicans* ATCC 10231™ (American type culture collection, Manassa, VA).

### Media and growth conditions

Yeast extract peptone (YEP) medium consisting of 1% (w/v) yeast extract, 2% (w/v) bacto-peptone (both from Difco Laboratories, Detroit, MI), 0.1% (w/v) KH_2_PO_4_, and 0.12% (w/v) NH_4_(SO_4_)_2_ was used as the basic medium supplemented with 2% (w/v) glucose (YPD) or 2% (w/v) glycerol (YPGly). The pH was adjusted to 5.5 with H_2_SO_4_ (10% w/v) before sterilization (115°C, 20 min). For growth of the WT-W303 strain, adenine 100 mg L^−1^ was added to the culture medium. For the *hem1Δ* mutant and/or for growth under anaerobiosis, the following anaerobic growth factors (AF) were added to the medium after sterilization (per liter): 15 mg ergosterol dissolved in 1 mL, Tween 80: pure ethanol (50:50, v/v).

The *yap1Δ*, *skn7Δ*, *ctt1Δ*, *sod1Δ*, *zwf1Δ*, *erg1Δ*, and *hem1Δ* mutants were precultivated in the presence of geneticin (G418 sulfate, 100 mg L^−1^). Aerobic or anaerobic precultures were performed at 30°C under shaking (180 rpm) in 100 mL Erlenmeyer flasks using a liquid to gas ratio of 1:10. Anaerobic precultures were incubated in anaerobic jars under an H_2_–CO_2_ atmosphere generated by Oxoid® gas-generating kits (Oxoid, Thermoscientific, Dardilly, France). Anaerobic Indicators (Oxoid®) were used to verify the absence of oxygen.

Aerobic cultures were performed in 24-well microtiter plates (Greiner, Frickenhausen, Germany). The initial optical density (OD_600 nm_) was 0.05 with an optical path length of 0.49 cm. At time zero, cells were treated with PYO (500 *μ*mol/L) by adding a small volume (2.5% v/v) of a stock solution (20 mmol/L) freshly prepared in methanol. Control wells were used to verify the absence of effect of the solvent alone (methanol). Cells were incubated for 24 h at 30°C under continuous stirring at 180 rpm in H_2_O-enriched air.

Anaerobic cultures were performed in 15 mL Hungate tubes filled with 3 mL YPD supplemented with AF. The culture medium was desaerated by bubbling pure sterile argon for 2 min prior inoculation at an initial OD_600 nm_ of 0.05. PYO (100–500 *μ*mol/L) was added and anaerobic conditions were reached by bubbling again pure argon for 2 min. Fermentation processes were carried out at 30°C and 180 rpm for 24 or 48 h.

For anaerobic growth of *C. albicans*, YPD medium was supplemented with 0.07% (w/v) L-cysteine and adjusted to pH 6.0 (Rosa et al. 2008). AF (ergosterol, tween 80) were added to the medium after sterilization.

### Chemicals

Purified PYO was purchased from Bertin Pharma (Montigny Le Bretonneux, France). G418 sulfate, H_2_O_2_, bovine superoxide dismutase (SOD) and catalase (CAT), potassium cyanide (KCN), antimycin A, Myxothiazol, HQNO (2-n-heptyl-4-hydroxyquinoline-N-oxide), L-ascorbate, tiron, resveratrol, L-cysteine hydrochloride monohydrate, and N-acetylcysteine (NAC) were purchased from Sigma-Aldrich (Saint-Quentin Fallavier, France).

Stock solutions were freshly prepared in distilled water or methanol or dimethylsulfoxide at appropriate concentrations. For each experiment, solvent controls were used.

### Cell-density measurements

Growth was monitored by measuring the optical density at 600 nm (OD_600 nm_) using 24-well microtiter plates (Greiner) and the FLUOstar OPTIMA microplate reader from (BMG Labtech GmbH, Ortenberg, Germany). Wells were filled with 1 mL of sample (optical path length = 0.49 cm). If needed, cultures and culture supernatants (controls) were diluted in saline water (NaCl 0.9% w/v). The final absorbance of PYO was systematically taken into account for cell-density determinations.

### Glucose and glycerol quantification

Glucose and glycerol were quantified using a Waters high pressure liquid chromatography system. Five microliters of filtered supernatant (on a 0.2 *μ*m pore filters) were loaded on a Phenomenex Rezex ROA-organic acid H^+^ column (300 by 7.8 mm) subjected to an isocratic method with 2.5 mmol/L H_2_SO_4_ as an eluant (flow rate of 0.5 mL min^−1^). Compounds were detected by a Waters differential refractometer. Products were identified by the use of glucose, glycerol, pyruvate, acetate, and lactate as external standard and concentrations were calculated using standard curves.

### Ethanol quantification

Ethanol quantification was performed on a Gas Chromatograph (Hewlett Packard model 5890 A; Hewlett Packard, Agilent Technologies France SAS, Les Ulis, France), equipped with a flame ionization detector (FID). The column used was an OV-1701 fused silica capillary column (25 m × 0.25 mm i.d. × 0.25 *μ*m film thickness; Chrompack, France). The split ratio was 43/2. The injector was kept at 220°C, and the detector was kept at 250°C. The column temperature was held at 50°C for 15 min. Nitrogen was used as carrier gas and the flow rate in the column was 2 mL min^−1^. Hydrogen and air were supplied to the FID at 38 and 398 mL min^−1^, respectively. 1-Propanol was used as internal standard.

### PYO quantification

The amount of PYO remaining in the rich medium after incubation was assessed spectrophotometrically at 690 nm, as previously reported (Price-Whelan et al. 2007). Culture supernatants incubated in the presence of PYO were vigorously reoxygenated until the 690 nm-signal stabilized. The absorbance of remaining oxidized PYO was then compared to the absorbance of 500 *μ*mol/L PYO freshly dissolved in culture supernatants incubated without PYO. Absorbance spectra from 300 to 720 nm were measured to verify more precisely the recovery of typical peaks in the 376 nm and 690 nm regions. Media pH values were adjusted at the same values before measurement.

### Cell viability

Cell viability was assessed by trypan blue staining. 200 *μ*L of cell suspension was mixed and incubated for 3 min at room temperature with an equal volume of 0.4% (w/v) trypan blue solution prepared in 0.81% (w/v) NaCl and 0.06% (w/v) dipotassium phosphate.

### Isolation of mitochondria and high-resolution oxygraphy

Mitochondria were isolated from the WT-W303 strain cultivated aerobically on YEP medium supplemented with adenine (100 mg L^−1^) and 2% (w/v) lactate. This carbon source was used as it allows the production of numerous mitochondria. Cells were harvested in the midexponential phase. Mitochondria were isolated using the procedure described by Avéret et al. (1998). Mitochondrial proteins were quantified by the Biuret method using bovine serum albumin (BSA) as standard.

For respiration measurements, mitochondria were resuspended at a final concentration of 0.1 mg protein mL^−1^ in the following buffer: mannitol 0.65 mol/L, ethylene glycol-bis aminoethylether N,N,N′,N′ tetraacetic acid 0.36 mmol/L, Tris/maleate 10 mmol/L (pH 6.7), BSA 0.3% (w/v). Oxygen concentrations were measured by high-resolution respirometry with the Oroboros Oxygraph-2k (Hutter et al. 2006) in standard configuration, with a 2 mL volume for the two chambers at 30°C and 500 rpm stirrer speed (Dufour et al. 2013). Data were recorded at 1 sec intervals using the Datlab 4 Acquisition software (Oroboros, Innsbruck, Austria). Standardized calibration procedures of the oxygen signal were carried out using the mannitol/Tris-maleate buffer. Respiration was automatically corrected for contributions of the polarographic oxygen sensor and of oxygen diffusion to total apparent respiration as a continuous function of oxygen concentration. P/O ratios (ATP formed vs. oxygen atoms consumed) were assessed by measuring the decrease in [O_2_] during the rapid burst of state 3 respiration after adding 0.1 mmol/L ADP. Oxygen consumption was expressed as pmoles of O_2_ consumed per second per unit of mitochondrial protein. Similar conditions were used for respiration measurements with whole cells, washed or not with Tris-maleate (100 mmol/L) buffer, KH_2_PO_4_ 10 mmol/L, pH 5.5. Data shown are the means (± SD) of six independent measurements resulting from two independent experiments.

## Results

### Oxidative stress only partially explains PYO toxicity

**Figure 2:**
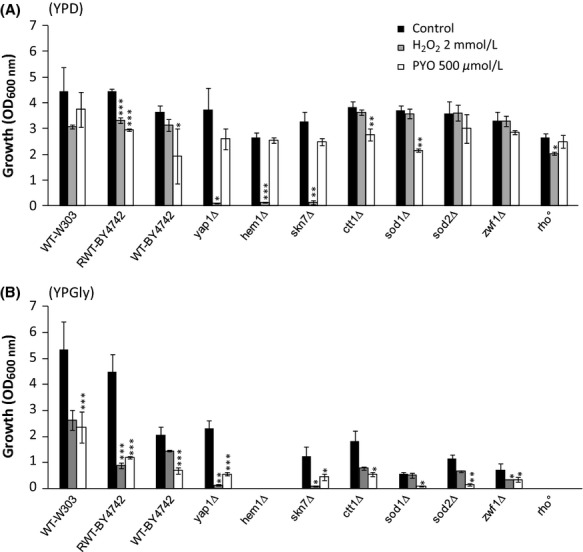
Effect of H_2_O_2_ and pyocyanin (PYO) on the aerobic growth (optical density [OD]_600 nm_) of several *Saccharomyces cerevisiae* strains. Yeast strains were cultivated aerobically for 24 h at 30°C on YPD medium (A) or on YPGly medium (B) in the presence of 2 mmol/L H_2_O_2_ or 500 *μ*mol/L PYO. Black bars correspond to the control experiments. OD_600 nm_ were measured with an optical path length of 0.49 cm. Cultures supernatants (diluted or not) were used as blank controls. Control wells were used to check the absence of effect of methanol 2.5% v/v (solvent of PYO). Results represent the means (± SD) of three to five separate experiments (12–20 absorbance values). Statistical significances of PYO effects (compared to the control) are indicated by the *P*-values: *P < 0.05; **P < 0.01; ***P < 0.001. Final pH values were about 4.6–4.9 and 6.0–7.1 in YPD and YPGly, respectively. Short description of the strains used (For further information, see Table S1 and *Saccharomyces genome database* at http://www.yeastgenome.org/): Wild-type W303 and BY-4742 are commonly used lab strains. WT-BY4742 is partially impaired in respiration as it is mutated in the HAP1 locus which encodes a major heme-responsive transcription factor that regulates O_2_-responsive genes. The RWT-BY4742 strain is restored for Hap1p (Gaisne et al. 1999). The *yap1Δ* and *skn7Δ* strains are hypersensitive to oxidative stress due to deletions of major transcription factors required for oxidative stress tolerance. The *hem1Δ* strain cannot synthesize heme and it is therefore unable to respire, devoid of catalase and cytochrome c peroxydase activities and impaired in lipid metabolism. The strains *ctt1Δ*, *sod1Δ, sod2Δ,* and *zwf1Δ* (directly derived from WT-BY4742) are deleted in cytosolic catalase, cytosolic Cu,Zn superoxide dismutase (SOD), mitochondrial Mn SOD, and cytosolic NADP(H)-dependent glucose-6-phosphate dehydrogenase, respectively. The *rho*^*0*^ strain is a respiratory-deficient mutant devoid of mitochondrial DNA.

PYO toxicity is generally attributed to the induction of oxidative stress (O'Malley et al. 2004a; Rada and Leto 2009; Muller 2011). The sensitivity of the different strains to oxidative stress was first assessed by measuring growth inhibition in the presence of 2 mmol/L H_2_O_2_, in YPD medium (Fig. 2A, gray bars). WT strains and certain mutants (described in Fig. 2 legend and Table S1) showed little, if any, sensitivity to 2 mmol/L H_2_O_2_. As expected, the *yap1Δ*, *hem1Δ*, and *skn7Δ* mutants were hypersensitive to H_2_O_2_.

In a parallel series, 500 *μ*mol/L PYO was added to YPD medium (Fig. 2A, white bars). PYO had a minor toxic effect on most strains, including the H_2_O_2_-hypersensitive *yap1Δ* and *skn7Δ* mutant strains. In addition, the *rho*^*0*^ mutant, which is devoid of the mitochondrial respiratory chain, was almost entirely resistant to PYO.

It is noteworthy that final pH values (4.6–4.9) were just below the pKa value of PYO (pKa = 4.9, O'Malley et al. 2004a). Nevertheless, we verified that the relative resistance of YPD-grown WT cells to PYO was pH-independent by using either alkalinized (pH 6.5)-, alkalinized (pH 6.5)-phosphate buffered (100 mmol/L)-or acidified-(pH 4.5) YPD media (data not shown).

These data suggest that the low-PYO toxic effects observed in certain strains are mediated in part by oxidative stress, however, caused by species other than H_2_O_2,_ and that additional phenomena are probably also involved. The data also suggest that respiration may reinforce PYO toxicity, as shown in bacteria (Hassan and Fridovitch 1980; Baron and Rowe 1981). YPD glucose-containing medium represses mitochondrial respiration and glucose is mainly catabolized by fermentation. We therefore studied the impact of H_2_O_2_ and PYO uniquely in oxidative conditions, using glycerol as growth substrate (YPGly medium). Under both YPD and YPgly conditions, PYO inhibited growth kinetics without affecting cell survival at any growth stage. Examples of growth kinetics are shown in Figure S1.

As expected, the respiratory-deficient *hem1Δ* and *rho*^*0*^ mutants did not grow on YPGly (Fig. 2B). All the mutant strains were growth impaired. Exposure to H_2_O_2_ caused greater growth inhibition for all the strains compared to similar conditions in YPD media, with the exception of *yap1Δ* and *skn7Δ* strains who were severely inhibited in both culture media (Fig. 2A and B, gray bars). Growth sensitivity was markedly increased (more than fivefold) for *ctt1Δ*, *sod2Δ,* and *zwf1Δ* mutants. In parallel, we examined PYO toxicity and found that the effect on growth was more than doubled compared to that observed on YPD medium for all the strains examined (Fig. 2B, white bars). Interestingly, the *sod1Δ* and *sod2Δ* mutants were found to be the most PYO-sensitive strains, suggesting the involvement of cytosolic and mitochondrial superoxide anions in PYO toxicity. However, little differences were found between the strains (hypersensitive or not to oxidative stress) in terms of response to PYO toxin. The data obtained using YPGly culture medium confirm the notion that actively respiring cells are more sensitive to PYO and that its effect is probably only partially due to oxidative stress.

### Antioxidants only slightly protect against PYO toxicity

**Figure 3:**
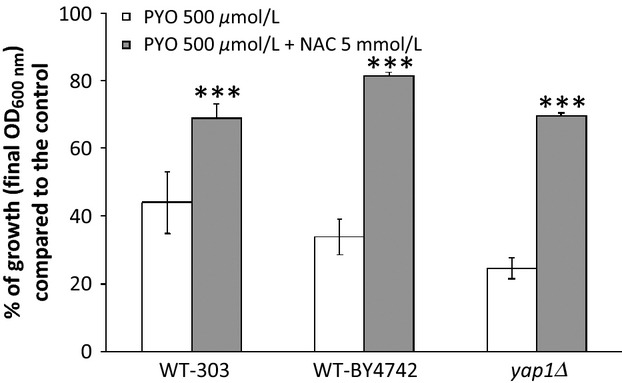
Protective effect of N-acetyl cystein against aerobic pyocyanin toxicity in WT-303, WT-BY4742 and *yap1Δ Saccharomyces cerevisiae* strains. Bars represent the percentages of growth observed compared to the controls. Yeast were cultivated aerobically for 48 h at 30°C on YPGly medium. Statistical significances of the NAC protective effects are indicated by the *P*-value: ***P < 0.001. All initial pH were adjusted to 5.5. Used alone, NAC had no effect on yeast growth (data not shown). The protective effect of NAC observed in WT-BY4742 (this figure) was similar in the complemented RWT-BY4742 strain and the *zwf1Δ* mutant (data not shown).

The addition of L-ascorbate (Muller 2009) or NAC can attenuate PYO toxicity in epithelial lung cells (Gloyne et al. 2011). We examined the effects of several cell-permeable antioxidants on PYO toxicity under oxidative conditions in YPGly medium. The antioxidants, L-ascorbate (10 mmol/L), resveratrol (200 *μ*mol/L), and tiron (2 mmol/L) had no effect or even slightly increased the inhibitory effect of PYO (data not shown). This suggests that the use of such antioxidants to counteract PYO toxicity needs to be reexamined. As shown in Figure 3, a protective effect was, however, observed in WT strains with NAC, a precursor of glutathione biosynthesis and a radical scavenger. A similar protective effect of NAC was found for the *yap1Δ* mutant that is hypersensitive to oxidative stress. The NAC effect was only partial and was identical for both WT and *yap1Δ* strains, which again suggests that PYO toxicity is due in part to an oxidative stress-independent process.

### PYO appears to interact with *S. cerevisiae* respiratory chain

**Figure 4:**
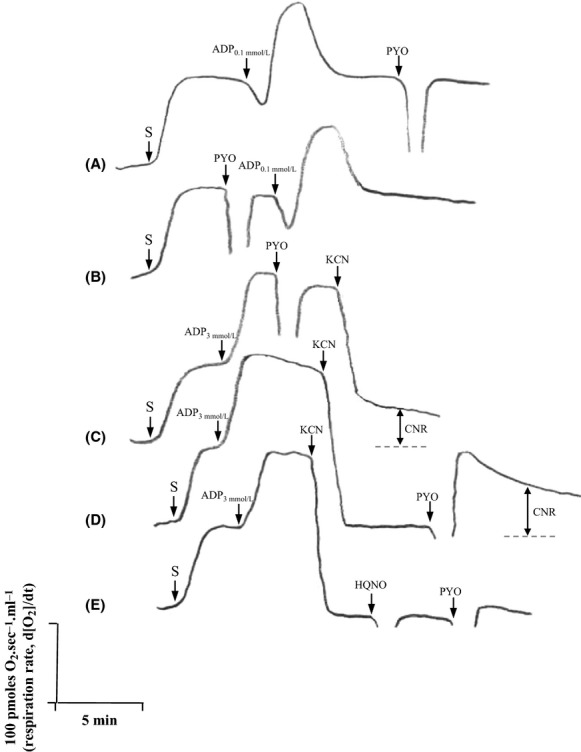
Effects of pyocyanin on respiration rates of isolated *Saccharomyces cerevisiae* mitochondria. O_2_ fluxes were measured by high-resolution respirometry (at [O_2_] up to 50 *μ*mol/L) by successive additions of substrate (S, succinate 7.5 mmol/L), ADP (0.1 or 3 mmol/L), pyocyanin (PYO 100 *μ*mol/L) and other poisons (potassium cyanide [KCN] 1 mmol/L and HQNO 20 *μ*mol/L^1^). Three separate experiments (six acquisitions) were performed but only typical acquisitions are shown. In (A and B), a limited amount of ADP (0.1 mmol/L) was added (conditions used for P/O determinations). In (C, D, and E),^2^ Effects were measured at state 3 respiration (excess of ADP, 3 mmol/L). CNR, cyanide-resistant respiration. ^1^Myxothiazol (10 *μ*mol/L) and antimycin A (10 *μ*mol/L) acted similarly to HQNO. ^2^Similar results were obtained in the absence of ADP (state 4) and/or with *α*-ketoglutarate as substrate.

We were unable to detect any effect of PYO (100–500 *μ*mol/L) on the instant respiration of YPGly-grown cells (data not shown). We, therefore, used isolated yeast mitochondria and examined whether PYO at 100 *μ*mol/L concentration interferes with the respiratory chain and whether a redox cycling occurs at this level. Succinate was added to intact mitochondria (Fig. 4). Our data suggest that the addition of PYO has no apparent effect on “state 4” respiration, that is, in the absence of ADP or after the conversion of ADP (100 *μ*mol/L) to ATP (Fig. 4A and B). It is possible that PYO alters the efficiency of ATP synthesis, therefore, we also measured the P/O ratios (ATP formed vs. oxygen atoms consumed). Similar P/O values were obtained with ADP (0.05 or 0.1 mmol/L) in the presence (1.64 ± 0.32) or absence (1.73 ± 0.33) of PYO. Moreover, the addition of PYO had no significant effect on the actively respiring “state 3” (excess of ADP, 3 mmol/L): specific mitochondrial O_2_ consumption rates were 1636 ± 61 and 1520 ± 175 pmoles O_2_ min^−1^ mg protein^−1^ in the absence or presence of PYO, respectively.

We also examined the effect of PYO together with two well-known respiratory poisons, cyanide and HQNO, also secreted by *P. aeruginosa* (Williams et al. 2007). An unusual cyanide-resistant respiration was detected (Fig. 4C). When PYO was added after cyanide (Fig. 4D), this cyanide-resistant respiration was again observed and was even stimulated. A peak of O_2_ consumption was seen just after the pulse of PYO but the signal stabilized after 8–10 min. As shown in Figure 4E, PYO did not induce this form of respiration in HQNO-poisoned mitochondria. Although no significant effect was detected on states 4 and 3 respirations, these results suggest that PYO can accept electrons from the respiratory chain through a complex III-dependent process.

In the actively respiring state 3, we assessed the release of ROS from mitochondria by adding CAT and/or SOD (both at 500 U/mL) after the consumption of 200 *μ*mol/L O_2_ and when the [O_2_] reached zero in the oxygraph chambers. We found that the addition of PYO (± cyanide) did not cause the release of detectable amounts of O_2_ originating from H_2_O_2_ or O_2_^·−^ (data not shown).

### PYO strongly affects growth of *S. cerevisiae* under anaerobiosis

Our data suggest that, in vivo, PYO could divert a minor part of the electron flow from the respiratory chain. This effect, as well as oxidative stress, could impede growth but both effects should vanish under anaerobiosis. Therefore, we tested the effect of PYO under fermentative anaerobic conditions, as has already been done for bacteria (Hassan and Fridovitch 1980; Baron and Rowe 1981).

**Figure 5:**
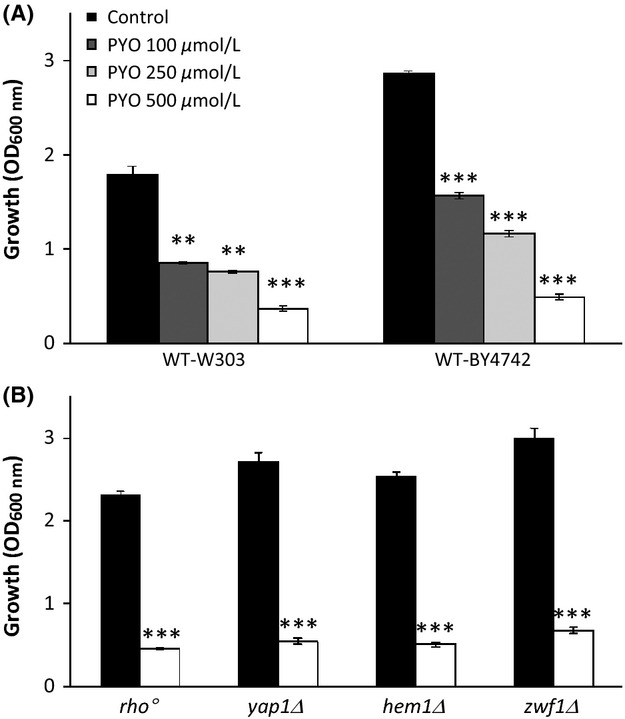
Effect of pyocyanin on the anaerobic growth of WT (A) and mutant strains (B) of *Saccharomyces cerevisiae*. Yeast were cultivated anaerobically for 24 h at 30°C in YPD medium supplemented with anaerobic growth factors, in the absence of pyocyanin (PYO) (black bars) or in the presence of PYO 100, 250, or 500 *μ*mol/L. Results represent the means ± SD of three separate experiments. Similar results were obtained with the RWT-BY4742 strain (83% inhibition with PYO 500 *μ*mol/L). The H_2_O_2_-hypersensitive trait of *yap1Δ* and *hem1Δ* mutants was also checked in anaerobic conditions (data not shown). Statistical significances of the pyocyanin effects are indicated by the *P*-values: **P < 0.01; ***P < 0.001.

Surprisingly, PYO (500 *μ*mol/L) was found to be highly toxic for yeast cells under anaerobiosis (Fig. 5). PYO toxicity (80% inhibition) increased fourfold for both WT-W303 and WT-BY4742 strains (Fig. 5A), compared to aerobic YPD conditions (Fig. 2A). Moreover, toxicity was also high (about 40% inhibition) with 100 *μ*mol/L PYO and was dose-dependent. PYO, at 100 or 500 *μ*mol/L concentrations, was fully reduced by yeast cells within a number of hours after the start of anaerobic growth. At 100 *μ*mol/L, PYO was colorless within less than 4 h (Fig. S2). At 500 *μ*mol/L, PYO was colorless at about 6 h (data not shown). Several control experiments were carried out in order to verify anaerobiosis and to clarify anaerobic PYO toxicity. We paid special attention to the potential involvement of trace amounts of O_2_. Yeast growth was not observed with glycerol as substrate. The *erg1Δ* mutant, which requires strict anaerobiosis to develop and therefore acted as a positive control, was readily able to grow under our conditions. In addition, anaerobic PYO toxicity was unaffected by NAC 5 mmol/L. These data (not shown) thus make it unlikely that there was an involvement of trace O_2_ in our anaerobic PYO toxicity measurements. We also verified that the effect of PYO was not influenced by the presence and composition of AF. The addition of AF and PYO did not increase toxicity under aerobiosis. The addition of 2 mg/L nicotinic acid (another AF, potentially present in limiting amounts in YPD) did not reduce anaerobic PYO toxicity (data not shown).

If anaerobic PYO toxicity is independent of ROS formation, the different mutants used previously should behave in a similar manner under anaerobiosis. This hypothesis was tested. The growth of the *yap1Δ*, *zwf1Δ*, and *hem1Δ* mutants in YPD medium exhibited comparable sensitivity to PYO as the WT strains (Fig. 5B). Interestingly the *rho*^*0*^ mutant, which has no mitochondrial DNA and for which the same behavior under aerobiosis and anaerobiosis should therefore be expected, was sensitive to PYO only under anaerobiosis. The presence of O_2_ may indirectly protect the cells from an alternative mechanism of PYO toxicity operating under anaerobiosis.

### PYO does not affect anaerobic fermentation parameters

We attempted to define, more precisely, the effects of PYO in anaerobic cells by examining several parameters during glucose fermentation (Table 1). After 48 h of growth, the cells incubated with PYO (500 *μ*mol/L) were almost all viable. This suggests that PYO affects anaerobic growth kinetics rather than killing the cells. As a consequence of growth inhibition, glucose consumption and ethanol production were strongly reduced in the presence of PYO. The yields of ethanol and glycerol production did not show major changes and no other by-products, such as pyruvate, could be detected in the culture medium. We conclude that the end-product formation pathways are probably not the site of action of PYO. No loss of PYO could be detected (aerobically) after 24 h (Fig. S3) and 48 h (data not shown) of anaerobic growth, suggesting that the effect is probably due to PYO itself and not to the accumulation of a degradation product. From these data, we hypothesize that a relatively high level of PYO radical is produced during anaerobic glucose fermentation and/or that this radical has higher toxicity than ROS.

**Table 1:** Effect of pyocyanin (PYO 500 *μ*mol/L) on anaerobic growth parameters.

Strains	WT-W303		WT-BY4742	
	−PYO	+PYO	−PYO	+PYO
Growth (final OD_600 nm_)^1^	1.78	0.39	2.66	0.365
Glucose consumed (moles)	111	63	110	47
Ethanol produced (moles)	177	102	186	73
Y ethanol/glucose	0.80	0.82	0.85	0.76
Glycerol produced (moles)	11.9	7.9	9.4	5.6
Y glycerol/glucose	0.054	0.063	0.043	0.059
Viability (%)	99	99	99	99
Final pH	4.99	5.3	4.93	5.02

Yeast cells were grown under anaerobiosis for 48 h at 30°C.

1 Measured with an optical path length of 0.49 cm. All the cultures were in stationary phase.

### PYO toxicity is not correlated to multidrug resistance

**Figure 6:**
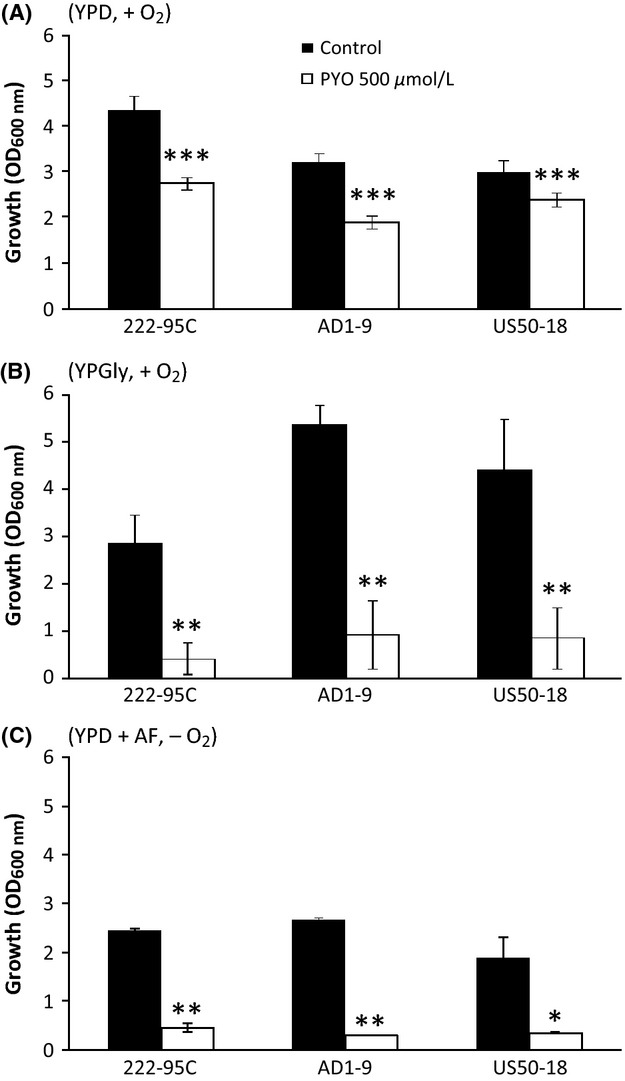
Effect of pyocyanin on the aerobic and anaerobic growth of WT-222-95C and mutant strains of *Saccharomyces cerevisiae*. AD1-9 strain has a decreased resistance to multiple drugs and US50-18 strain has an increased resistance to drugs. Yeast were cultivated at 30°C aerobically for 24 h on YPD medium (A) and for 48 h on YPGly (B) or for 24 h on YPD supplemented with anaerobic growth factors (C). Statistical significances of the pyocyanin effects are indicated by the *P*-values: *P < 0.05; **P < 0.01; ***P < 0.001.

We asked the question whether the high toxicity of PYO under anaerobiosis could be explained by a decrease in the expression of ABC transporters involved in multidrug resistance. We compared the effects of PYO, under aerobic and anaerobic conditions, on WT-222-95-C strain and both AD1-9 and US50-18C mutant strains. AD1-9 strain carries multiple deletions in 7 ABC genes (YOR1, SNQ2, PDR5, YCF1, PDR10, PDR11, and PDR15) and in two transcription activation factors (PDR1 and PDR3). These mutations render the cells 2–200 times more sensitive to numerous toxic compounds including small heterocyclic compounds such as resazurin and 8-hydroxyquinoline that share some similarities with PYO (Rogers et al. 2001; Decottignies et al. 2002). The US50-18C strain carries the pdr1-3 activating mutation that confers considerable multiple drug resistance. As shown in Figure 6, the three strains exhibited similar patterns of PYO inhibition. Toxic effects on yeast growth were low during aerobic fermentation (YPD), especially in the case of the US50-18C strain. Toxicity was increased significantly during aerobic respiration (YPGly) and anaerobic fermentation (YPD + AF) for all strains tested. These data suggest that pleiotropic drug resistance-related *ABC* transporters in the yeast *S. cerevisiae* are not involved in PYO toxicity.

### PYO toxicity is not linked to DNA intercalation

PYO binds directly and intercalates the DNA (Hollstein and Van Gemert 1971; Das et al. 2013). Although DNA intercalation occurs via noncovalent interactions and is reversible, PYO could cause substantial changes in DNA structure under our conditions. The potential link between anaerobic toxicity and DNA damage was therefore explored using *S. cerevisiae* mutants, which are deficient in homologous recombination and DNA damage repair (nucleotide or base excision repair). Experiments were carried out as for Figures 2, 5. As shown in Figure S4, the mutants did not show increased sensitivity to PYO, when 500 and 100 *μ*mol/L PYO were added under aerobic and anaerobic conditions, respectively. Under these conditions, no link was found between PYO toxicity and DNA damage.

### *Candida albicans* demonstrates similar PYO sensitivity under anaerobiosis to *S. cerevisiae*

**Figure 7:**
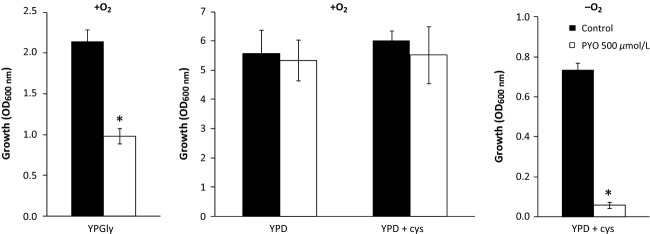
Effect of pyocyanin 500 *μ*mol/L on the aerobic and anaerobic growth of *Candida albicans*. Yeast were cultivated at 30°C aerobically for 24 h on YPGly, YPD or YPD supplemented with 0.07% (w/v) L-cysteine and anaerobically for 48 h on YPD supplemented with 0.07% (w/v) L-cysteine and anaerobic growth factors. Statistical significance of the pyocyanin (PYO) effect is indicated by the *P*-value: *P < 0.05.

We examined whether PYO can affect the anaerobic growth of another species of yeast. The pathogenic crabtree-negative yeast *C. albicans* was chosen because this fungus, together with *Aspergillus fumigates*, may coexist with *P. aeruginosa* in the lungs of cystic fibrosis (CF) patients (McAlester et al. 2008). This species is capable of anaerobic growth in the presence of L-cysteine (Rosa et al. 2008) and AF. Importantly, we found that high anaerobic PYO toxicity was also elicited in *C. albicans* (Fig. 7). PYO toxicity was lower under aerobiosis in the presence or absence of L-cysteine.

Altogether, these data strongly suggest that PYO is an anaerobic poison.

## Discussion

As recently discussed by Okegbe et al. (2012), the understanding of the biological roles of redox-active metabolites such as the phenazine PYO requires characterization of their specific biological effects in a condition-dependent manner. Numerous studies have reported the toxic effects of PYO. However, to our knowledge, the relationships between PYO toxicity, respiratory capacities, oxidative stress, and O_2_ tension have not yet been examined in a eukaryotic organism. The interaction of PYO with mitochondrial respiration has only been documented to a small extent (Friedheim 1931; Armstrong and Stewart-Tull 1971; O'Malley et al., 2003b). In this study, we first reexamined PYO toxicity using the standard medium (YPD) for growth of several *S. cerevisiae* strains. For these experiments, we used concentrations of PYO (500 *μ*mol/L ≡ 105 *μ*g/mL) that are similar to the concentrations previously tested in *S. cerevisiae* by other authors (Ran et al. 2003 [25–100 *μ*g/mL]; Angell et al. 2006 [25–250 *μ*g/mL]). These concentrations are higher than the concentration of PYO detected in infected lungs of CF patients (Wilson et al. 1988; Hunter et al. 2012). Notwithstanding, a relative toxin resistance to 500 *μ*mol/L PYO was observed for several genetic backgrounds. We verified that this was not caused by acidification of the medium that could modify the color and protonation state of PYO at pH ≤ 4.9. For the first time, we provide data on PYO toxicity in actively respiring *S. cerevisiae* cells (in YPGly medium) and during strict fermentation (anaerobiosis). We found that oxidized PYO (100 *μ*mol/L) did not affect respiration of yeast cells or of isolated mitochondria. However, using cyanide and HQNO (and other complex III inhibitors), we found that PYO could interfere with mitochondrial respiration at the level of complex III or of a downstream target. PYO can, therefore, be considered as another mitochondria-targeted agent, together with the well-known poisons, cyanide and HQNO, also produced by *P. aeruginosa* under hypoxia (Machan et al. 1992; Williams et al. 2007). Despite the apparent absence of a short-term effect of PYO on cellular and “state 3” O_2_ fluxes, it is still unknown whether a futile redox cycling of PYO occurs in vivo in mitochondria. In this study, we obtained a panel of data showing a role for mitochondrial respiration in triggering aerobic PYO toxicity. PYO might also target specific steps in the oxidative metabolism of glycerol (e.g., glycerol-3-phosphaste dehydrogenase).

Despite the inhibition of PYO toxicity by NAC, the role of oxidative stress remains unclear. For instance, the *yap1Δ* and *skn7Δ* mutants, both of whom are hypersensitive to oxidative stress, were not PYO-hypersensitive.

The most striking result of this study is the high toxicity observed with PYO under anaerobiosis (argon atmosphere). Increased toxicity of PYO under anaerobiosis has already been observed in bacteria under denitrifying conditions, but not under fermentative conditions (Baron and Rowe 1981). The authors concluded that active respiration, but not necessarily the presence of O_2_, was required for PYO toxicity and that under anaerobiosis, a nitrogen radical instead of ROS could be involved. They also proposed that PYO could divert electron flow at a specific site of the respiratory chain, thus disturbing oxidative phosphorylation. As recently shown in *Escherichia coli* by Gu and Imlay (2011), redox drugs such as phenazine methosulfate (PMS) quickly accumulate in a reduced inactive form during anaerobic fermentation (when oxygen is unavailable to reoxidize them). The anaerobic toxicity of PMS requires its reoxidation by a terminal electron acceptor in the respiratory chain. In both *S. cerevisiae* and *C. albicans* (this work), anaerobic PYO toxicity is entirely independent of the respiratory chain, as demonstrated here with *rho* and *hem1*Δ mutants. Recently, Imlay (2013) pointed out that, even under anoxic conditions, redox-cycling compounds can be toxic because of their ability to destabilize Fe-S cluster containing enzymes, especially not only dehydratases involved in amino acid biosynthesis but also aconitase and fumarase. In *S. cerevisiae*, PYO inhibited anaerobic growth without any change in cell viability and without a significant effect on the apparent redox balance. We hypothesize a mechanism similar to Imlay to explain the anaerobic PYO toxicity in *S. cerevisiae*. In the absence of O_2_ and at high fermentative glycolytic fluxes (Gancedo et Serrano, 1989; Beauvoit et al., 1993), the cross-reaction between NAD(P)H-reduced and oxidized PYO could lead to elevated levels of the radical form (Fig. 1). We propose that this form reacts with Fe-S-dependent enzymes and impairs growth more efficiently than the ROS formed under aerobiosis. To solve some of these questions, specific electronic paramagnetic resonance (EPR) experiments are currently being designed to quantify the PYO radical, in both aerobic and anaerobic *S. cerevisiae* cells. This approach will then be extended to *C. albicans*, other fungi, and to mammalian cells under transient anaerobiosis.

Certain bacteria and fungi are susceptible to PYO and other phenazines at low concentrations (*μ*mol/L range) (Hassan and Fridovitch 1980; Baron and Rowe 1981). This is not the case in *S. cerevisiae*. 100 *μ*mol/L were required for 50% inhibition of anaerobic growth. This difference reinforces the idea that there are probably many different mechanisms of PYO toxicity in living organisms. Nevertheless, it should be kept in mind that the concentration used in anaerobiosis (100 *μ*mol/L) is of the same order as those observed (˜10–130 *μ*mol/L or 25–50 *μ*mol/L) in the lungs of CF patients infected by *P. aeruginosa* (Wilson et al. 1988; Hunter et al. 2012).

PYO synthesis helps *P.aeruginosa*'s growth and survival under O_2_-limitations (Price-Whelan et al. 2007; Wang et al. 2010). In addition, O_2_-limitations are encountered in vivo by eucaryotic target cells: during cystic fibrosis (CF), *P. aeruginosa* actively produces a microaerobic environment even at high aeration rates due to the overproduction of alginate (Sabra et al. 2002). Very steep hypoxic gradients or even anaerobic conditions can be generated in the airway mucus of CF patients (Stutts et al. 1986; Worlitzsch et al. 2002; Yoon et al. 2002; Hassett et al. 2009). In this type of anaerobic microenvironment, target host cells or infectious agents (*C. albicans* or other fungi) may be exposed to increased PYO toxicity. Taking into account these data and the results of this study, further research is clearly needed to fully understand the impact of hypoxia and anoxia on PYO toxicity and on *P. aeruginosa* pathogenicity.

## Supporting Information

**Figure S1.** Typical aerobic growth kinetics of *Saccharomyces cerevisiae* strains in the absence (▪) or presence (□) of PYO 500 *μ*gmol/L. W303 (A), BY-4742 (B) *hem↿* (C) *yap↿* (D) and *rho^0^* (E) strains were grown at 30°C on YPD medium. W303 (F) BY-4742 (G) *yap↿* (H) strain were grown at 30°C on YPGly medium. Results are the means of four determinations. For the control experiments (▪), we checked the absence of effect of the solvent alone (methanol). Using trypan blue, we also checked that PYO did not increase cell mortality (at detectable levels) and did not induce significant changes in yeast cell morphology at any growth stage (data not shown). Even minor on YPD medium, PYO toxicity was visible in middle or late growth stages. In the particular *hem↿* mutant (C) cumulating several severe dysfunctions (respiratorydeficiency, strong impairments of lipid metabolism and of O_2_ and ROS adaptations), growth was delayed in the presence of PYO but final growth (after 24 h) was unaffected (see also Fig. 22A). **Figure S2.** Typical photographs showing the decoloration of pyocyanin (PYO) during anaerobic cultures in Hungate tubes. In this example, the WT-303 strain was used. The culture medium was YPD supplemented with anaerobic growth factors. At *t* = 0, OD_600 nm_ was 0.05 (*l* = 0.49 cm). Depending on the initial concentration, PYO became colorless after a few hours of cultivation. At time 4 h, PYO 100 *μ*mol/L was colorless while PYO 500 *μ*mol/L was only partially discolored. At time 24 h, the blue color of PYO 500 *μ*mol/L was entirely recovered after a vigorous oxygenation of the tube. PYO recovery was also measured by spectrophotometry as described in Material and Methods (Fig. S3 and data not shown). **Figure S3.** Assessment of the amount of pyocyanin (PYO) remaining in the rich medium after 24 h of anaerobic growth (WT-BY4742 strain). Culture supernatants incubated in the presence of PYO 500 *μ*mol/L were vigorously reoxygenated until the 690 nm-signal stabilized. The absorbance of the remaining oxidized PYO was then compared to the absorbance of 500 *μ*mol/L pyocyanin freshly dissolved in culture supernatants incubated without pyocyanin. Media pH were adjusted at the same values before measurement. Three comparative assays were performed. **Figure S4.** Effect of pyocyanin 500 *μ*mol/L and 100 *μ*mol/L on the aerobic and anaerobic growth of WT-BY4742 and mutant strains of *Saccharomyces cerevisiae* deficient in DNA damage repair ability. Yeast were cultivated at 30°C aerobically for 24 h on YPD medium (A) and for 48 h on YPGly medium (B) or anaerobically for 24 h on YPD supplemented with anaerobic growth factors (C). Rad mutants (derived from WT-BY4742) are affected in nucleotide excision repair or recombinational repair of double-strand breaks in DNA. The mutant lar013 (derived from the control strain lar009), deleted in *Ntg1, Ntg2* and *Apn1* genes, is affected in base excision repair and repair of DNA damage caused by oxidation and alkylating agents (see Table S1 for further details).

**Table S1.** *Saccharomyces cerevisiae* strains used in this study.
