# Supplementary material for: Unsuspected pyocyanin effect in yeast under anaerobiosis
Source: Microbiologyopen. 2013 Dec 5;3(1):1–14. doi: 10.1002/mbo3.142 (PMC3937724; doi:10.1002/mbo3.142)
Supplement: Supplementary file 1 — Figure S1. Typical aerobic growth kinetics of Saccharomyces cerevisiae strains in the absence (▪) or presence (□) of PYO 500 μgmol/L. W303 (A), BY-4742 (B) hem↿ (C) yap↿ (D) and rho0 (E) strains were grown at 30°C on YPD medium. W303 (F) BY-4742 (G) yap↿ (H) strain were grown at 30°C on YPGly medium. Results are the means of four determinations. For the control experiments (▪), we checked the absence of effect of the solvent alone (methanol). Using trypan blue, we also checked that PYO did not increase cell mortality (at detectable levels) and did not induce significant changes in yeast cell morphology at any growth stage (data not shown). Even minor on YPD medium, PYO toxicity was visible in middle or late growth stages. In the particular hem↿ mutant (C) cumulating several severe dysfunctions (respiratorydeficiency, strong impairments of lipid metabolism and of O2 and ROS adaptations), growth was delayed in the presence of PYO but final growth (after 24 h) was unaffected (see also Fig. 22A). Figure S2. Typical photographs showing the decoloration of pyocyanin (PYO) during anaerobic cultures in Hungate tubes. In this example, the WT-303 strain was used. The culture medium was YPD supplemented with anaerobic growth factors. At t = 0, OD600 nm was 0.05 (l = 0.49 cm). Depending on the initial concentration, PYO became colorless after a few hours of cultivation. At time 4 h, PYO 100 μmol/L was colorless while PYO 500 μmol/L was only partially discolored. At time 24 h, the blue color of PYO 500 μmol/L was entirely recovered after a vigorous oxygenation of the tube. PYO recovery was also measured by spectrophotometry as described in Material and Methods (Fig. S3 and data not shown). Figure S3. Assessment of the amount of pyocyanin (PYO) remaining in the rich medium after 24 h of anaerobic growth (WT-BY4742 strain). Culture supernatants incubated in the presence of PYO 500 μmol/L were vigorously reoxygenated until the 690 nm-signal stabilized. The absorbance of the remaini [file mbo30003-0001-sd1.rtf]

  
Figure S1. Typical aerobic growth kinetics of S. cerevisiae strains in the absence () or presence () of PYO 500 µM.  W303 (A), BY-4742 (B) hem1D (C) yap1D (D) and rho0 (E) strains were grown at 30°C on YPD medium. W303 (F) BY-4742 (G) yap1D (H) strain were grown at 30°C on YPGly medium.
Results are the means of 4 determinations. For the control experiments (), we checked the absence of effect of the solvent alone (methanol). Using trypan blue, we also checked that PYO did not increase cell mortality (at detectable levels) and did not induce significant changes in yeast cell morphology at any growth stage (data not shown). Even minor on YPD medium, PYO toxicity was visible in middle or late growth stages. In the particular hem1D mutant (C) cumulating several severe dysfunctions (respiratory-deficiency, strong impairments of lipid metabolism and of O2 and ROS adaptations), growth was delayed in the presence of PYO but final growth (after 24 h) was unaffected (see also Fig. 2A).


Figure S2. Typical photographs showing the decoloration of pyocyanin (PYO) during anaerobic cultures in Hungate tubes. In this example, the WT-303 strain was used. The culture medium was YPD supplemented with anaerobic growth factors. At t=0, OD600nm was 0.05 (l=0.49cm). Depending on the initial concentration, PYO became colorless after a few hours of cultivation. At time 4 h, PYO 100 µM was colorless while PYO 500 µM was only partially discolored. At time 24 h, the blue color of PYO 500 µM was entirely recovered after a vigorous oxygenation of the tube. PYO recovery was also measured by spectrophotometry as described in Material & Methods (Fig. S3 and data not shown).


                   
Figure S3. Assessment of the amount of pyocyanin (PYO) remaining in the rich medium after 24 h of anaerobic growth (WT-BY4742 strain).
Culture supernatants incubated in the presence of PYO 500 µM were vigorously re-oxygenated until the 690 nm-signal stabilized. The absorbance of the remaining oxidized PYO was then compared to the absorbance of 500 µM pyocyanin freshly dissolved in culture supernatants incubated without pyocyanin. Media pH were adjusted at the same values before measurement. Three comparative assays were performed. 


                   
Figure S4. Effect of pyocyanin 500µM and 100 µM on the aerobic and anaerobic growth of WT-BY4742 and mutant strains of S. cerevisiae deficient in DNA damage repair ability.
Yeast were cultivated at 30°C aerobically for 24 h on YPD medium (A) and for 48 h on YPGly medium (B) or anaerobically for 24 h on YPD supplemented with anaerobic growth factors (C). Rad mutants (derived from WT-BY4742) are affected in nucleotide excision repair or recombinational repair of double-strand breaks in DNA. The mutant lar013 (derived from the control strain lar009), deleted in Ntg1, Ntg2 and Apn1 genes, is affected in base excision repair and repair of DNA damage caused by oxidation and alkylating agents (see Table S1 for further details).
