# Supplementary material for: Unsuspected pyocyanin effect in yeast under anaerobiosis
Source: Microbiologyopen. 2013 Dec 5;3(1):1–14. doi: 10.1002/mbo3.142 (PMC3937724; doi:10.1002/mbo3.142)
Supplement: Supplementary file 2 — Table S1. Saccharomyces cerevisiae strains used in this study. [file mbo30003-0001-sd2.rtf]

Table S1. S. cerevisiae strains used in this study.
Strain	Genotype	Characteristics	Origin/Reference	
WT-W303-1B        	MATá, leu2-3,112, ade2-1, his3-11,15, trp1-1, ura3-1, can1-100	WT strain	R.Rothstein (Columbia University, NY)	
WT-BY47421	MATa, his3D1, leu2D0, lys2D0, ura3D0 		WT strain derived from S288c carrying a defective Ty1 element inserted in the 3' region of the HAP1 ORF encoding a haem-responsive transcription factor that regulates gene expression in response to O2. WT strain partially impaired in respiration1.	EUROSCARF (Y10000)
Gaisne et al, 1999	
RWT-BY47421	WT BY4742 transformed by an URA3-centromeric plasmid (pFL38) carrying the wild-type HAP1 gene.
	WT BY4742 strain restored for HAP1, and therefore restored for cytochrome c levels and respiration1.
	Gift from Anne Devin (CNRS, Bordeaux). Plasmid provided by Jacqueline Verdière (CNRS, Paris). Gaisne et al, 1999.	
Mutant strains derived from WT-BY4742 MATa (or WT-BY4741 MATa)	
rho°                                                                    	No mitochondrial DNA. Obtained from WT-BY4742 by mutagenesis with ethidium bromide.	Absence of mitochondrial respiration. 	This study
	
yap1D	MATa, his3D1, leu2D0, met15D0,  ura3D0, YML007w::kanMX4	Deleted in the major Basic leucine Zipper (bZIP) transcription factor required for oxidative stress tolerance. Mutant hypersensitive to oxidative stress.	EUROSCARF (Y00569)	
skn7D  	MATa, his3D1, leu2D0, lys2D0 ura3D0, YHR206w::kanMX4	Deleted in the transcription factor required for optimal induction of heat-shock genes in response to oxidative stress. Mutant hypersensitive to oxidative stress.	EUROSCARF (Y12900)	
ctt1D	MATa, his3D1, leu2D0, lys2D0, ura3D0, YGR088w::kanMX4	Deleted in the cytosolic catalase.	EUROSCARF (Y14718)	
sod1D	MATa, his3D1, leu2D0, lys2D0, ura3D0, YJR104c::kanMX4	Deleted in the cytosolic Cu-Zn superoxide dismutase. 	EUROSCARF (Y16813)	
Sod2D	MATa, his3D1, leu2D0, lys2D0, ura3D0, YHR008c::kanMX4	Deleted in the mitochondrial Mn superoxide dismutase. 	EUROSCARF (Y16605)	
zwf1D                                  	MAT a, his3D1, leu2D0, lys2D0,  ura3D0, YNL241c::kanMX4
	Deleted in the glucose-6-P dehydrogenase (first step of pentose phosphate pathway). Strain partially impaired in NADPH regeneration and oxidative stress resistance.	EUROSCARF (Y11971)
Minard & McAlister, 2001	
hem1D
(TBY23591)	MATa, YDR232w::kanMX4, obtained from Y23591 MATa/a (EUROSCARF) by random spore analysis3
	Deleted in the 5-aminolevulinate synthase which catalyzes the first step of the haem biosynthetic pathway. Mutant  impaired in the regulation of O2-responsive genes and lipid metabolism, and devoid of functional cytochromes, catalases and other haem-containing enzymes2. Unable to respire.	This study
Dawes & Hardie, 1974 (random spore analysis)	
erg1D
(TBY24805)	MATa, YGR175C::kanMX4, obtained from Y24805 mat a/a (EUROSCARF) by random spore analysis3	Deleted in the squalene epoxidase, the first O2-dependent step of ergosterol biosynthesis. Grows only under strict anaerobic conditions2.	This study
Dawes & Hardie, 1974 (random spore analysis)
	
Other strains 	
WT-222-95C	MATa, PDR1, ura3 (isogenic to Ó1278b)	WT strain	Rogers et al (2001)
Decottignies et al (2001)	
US50-18C	MATá, pdr1-3, ura3, his1, obtained by cross between DRI9-T8 and WT-222-95C	The pdr1-3 allele encodes a hyperactive mutated form of Pdr1p, resulting in increased levels of the ABC transporter proteins. Therefore, this strain is resistant to multiple drugs.	Rogers et al (2001)
Decottignies et al (2001)	
AD1-9 (AD123456789)-	Derived from US50-18C.
MATá, ura3, his1, yor1Ä::hisG, snq2Ä::hisG, pdr5-Ä2::hisG, pdr10Ä::hisG, pdr11Ä::hisG, ycf1Ä::hisG, pdr3-Ä2::hisG, pdr15Ä::hisG, pdr1-Ä3::hisG	This strain contains multiple gene deletions of transcription factors and membrane-associated ABC transporters involved in pleiotropic drug resistance.	Rogers et al (2001)
Decottignies et al (2001)	
1 The partial defect in respiration of the WT-BY4742 strain was confirmed by comparing its specific cellular oxygen consumption capacities and biomass yields (about 50 % reduced) with those of the RWT-BY4742 strain in regulated-batch YPD cultures (unpublished data). 2 Rosenfeld & Beauvoit (2003) for review. 3 Dawes & Hardie (1974)


Table S1 (continued)
Strain	Genotype	Characteristics	Origin/Reference	
Mutant strains affected in DNA damage repair (derived from WT-BY4742 MAT a or hDNP42)	
Rad1D
	(WT-BY4742) MATa, his3D1, leu2D0, lys2D0 ura3D0,
YPL022w::kanMX4
	Deleted in single-stranded DNA endonuclease; Rad1p cleaves single-stranded DNA during nucleotide excision repair and double-strand break repair; subunit of Nucleotide Excision Repair Factor 1 (NEF1); homolog of human XPF protein	EUROSCARF (Y12806)
(www.yeastgenome.org)	
Rad2D
	(WT-BY4742) MATa, his3D1, leu2D0, lys2D0 ura3D0,
YGR258c::kanMX4 
	Deleted in single-stranded DNA endonuclease; Rad2p cleaves single-stranded DNA during nucleotide excision repair to excise damaged DNA; subunit of Nucleotide Excision Repair Factor 3 (NEF3); homolog of human XPG protein	EUROSCARF (Y17289)
(www.yeastgenome.org)	
Rad51D
	(WT-BY4742) MATa, his3D1, leu2D0, lys2D0, ura3D0,
YER095w::kanMX4
	Deleted in a strand exchange protein; Rad51p forms a helical filament with DNA that searches for homology; involved in the recombinational repair of double-strand breaks in DNA during vegetative growth and meiosis; homolog of Dmc1p and bacterial RecA protein	EUROSCARF (Y16401)
(www.yeastgenome.org)	
Rad52D
	(WT-BY4742) MATa, his3D1, leu2D0, lys2D0 ura3D0,
 YML032c::kanMX4
	Deleted in a protein that stimulates strand exchange by facilitating Rad51p binding to single-stranded DNA; Rad52p anneals complementary single-stranded DNA; involved in the repair of double-strand breaks in DNA during vegetative growth and meiosis	EUROSCARF (Y10540)
(www.yeastgenome.org)	
LAR009	MATa, his7-l, lys2A5'::LEU-lys2A3', ade5-l, trpl-289, ura3-52	Control strain (when compared to LAR013).
Haploid isogenic derivative of heterozygous diploid hDNP42 	Rowe et al (2012)
(www.yeastgenome.org)	
LAR013	MATa, his7-l, lys2A5'::LEU-lys2A3', ade5-l, trpl-289, ura3-52, ntgl::hphMX4, ntg2::BSD, apnl::TRPl	Haploid derivative of heterozygous diploid hDNP42,deleted in:
Ntg1p: DNA N-glycosylase and apurinic/apyrimidinic lyase; involved in base excision repair; acts in both nucleus and mitochondrion; creates a double-strand break at mtDNA origins that stimulates replication in response to oxidative stress; required for maintaining mitochondrial genome integrity; NTG1 has a paralog, NTG2, that arose from the whole genome duplication

Ntg2p (NTG2 is a paralog of NTG1): involved in base excision repair, localizes to the nucleus.

Apn1p: major apurinic/apyrimidinic endonuclease, 3'-repair diesterase involved in repair of DNA damage by oxidation and alkylating agents; also functions as a 3'-5' exonuclease to repair 7,8-dihydro-8-oxodeoxyguanosine; genetically interacts with NTG1 to maintain mitochondrial genome integrity	Rowe et al (2012)
(www.yeastgenome.org)	
